# A Comparison of the Costs of Managing Proximal Humerus Fractures in a Cohort of Patients Injured in Road Traffic Incidents Under the Transport Accident Commission Scheme in the State of Victoria

**DOI:** 10.1111/ans.70669

**Published:** 2026-04-09

**Authors:** Filip Cosic, Elton Edwards, Richard Page, Lara Kimmel, Belinda Gabbe

**Affiliations:** ^1^ Department of Orthopaedic Surgery The Alfred Melbourne Australia; ^2^ School of Public Health and Preventive Medicine Monash University Melbourne Australia; ^3^ Department of Orthopaedic Surgery University Hospital Geelong Geelong Australia; ^4^ Barwon Centre for Orthopaedic Research and Education (B‐CORE) St John of God Hospital and Deakin University Geelong Victoria Australia; ^5^ Department of Physiotherapy The Alfred Melbourne Australia; ^6^ Health Data Research UK Swansea University Medical School, Swansea University Swansea UK

**Keywords:** costs and cost analysis, internal fixation, proximal humeral fracture, replacement shoulder arthroplasty, shoulder fractures

## Abstract

**Background:**

Proximal humerus fractures are becoming more prevalent with associated increases in operative management. This study aimed to compare costs between operative and non‐operative management of these fractures in an Australian population.

**Methods:**

A cost‐analysis was performed in a cohort of patients initially managed at a Level 1 Trauma Centre between January 2010 and December 2018, who sustained a proximal humerus fracture following a road traffic injury. Patients were identified using ICD‐10‐AM coding and matched to the Victorian Orthopaedic Trauma Outcome Registry. This cohort was linked with data from the state's no‐fault third‐party insurer for transport injuries to obtain total claims cost data for the acute admission, subsequent admissions, and rehabilitation costs in Australian dollars.

**Results:**

A total of 113 patients met the inclusion criteria; 31 non‐operative and 81 operative. The mean (SD) age was 50.1 (20.3) years in the non‐operative group and 44.0 (15.8) in the operative group (*p* = 0.10). In the non‐operative group, 87% of patients sustained other injuries compared with 76% of patients in the operative group (*p* = 0.30). The median total treatment cost in the non‐operative group was A$48 982 compared to A$36 457 in the operative group (*p* = 0.99). Median subsequent admission costs for the respective groups were A$9 643 and A$8 378 (*p* = 0.77) and median rehabilitation costs were A$6 016 and A$4 754 respectively (*p* = 0.95).

**Conclusion:**

Healthcare associated costs did not differ significantly in trauma patients with proximal humerus fractures regardless of management undertaken. Concerns regarding healthcare costs should not impact on surgical decision‐making when managing these fractures.

## 1 Introduction

Proximal humerus fractures remain one of the most common orthopaedic injuries, occurring in a bimodal distribution with younger patients typically sustaining fractures following higher energy injuries, in contrast to older patients where low falls are the most common cause [1, 2]. Whilst there is increasing evidence to support non‐operative management in the majority of patients, rates of operative intervention have continued to increase [3, 4, 5, 6]. Notably there has been a shift in the surgical management of these injuries, secondary to high rates of failure with internal fixation [7, 8, 9, 10], with the increasing use of reverse shoulder arthroplasty, particularly in older adults [5, 6, 11, 12].

The increasing rates of shoulder arthroplasty, whilst associated with lower complication rates, have led to increasing costs in the management of these injuries [11, 13, 14]. Shi et al. found increasing rates of arthroplasty being performed to manage proximal humerus fracture, with associated higher costs of management compared to internal fixation [11]. Similarly, Manoli et al. found increased costs with shoulder arthroplasty for managing proximal humerus fractures, despite shorter lengths of stay [13]. Whilst multiple studies evaluating the cost of these injuries have been performed, these studies have focused solely on initial in hospital costs, with no data available on rehabilitation and subsequent readmission costs. Further studies have been conducted in predominantly European nations and the United States of America, with no studies evaluating the cost of managing these injuries undertaken in an Australian population [11, 13, 14, 15, 16, 17, 18].

The aim of this study was to describe and compare the in‐hospital, subsequent admission, and rehabilitation costs of managing proximal humerus fractures in an Australian road transport‐related injury population.

## 2 Methods

### 2.1 Setting and Participants

This observational study was conducted at a Level 1 Trauma centre and included patients presenting between January 2010 and December 2018 with a proximal humerus fracture resulting from a road transport‐related cause. Eligible patients were identified using the relevant International Statistical Classification of Diseases and Related Health Problems, Tenth Revision, Australian Modification (ICD‐10‐AM) coding (code S42.2—fracture of upper end of humerus) [19]. Radiographs were then screened to confirm proximal humerus fractures had been sustained. Patients were subsequently matched with the Victorian Orthopaedic Trauma Outcomes Registry (VOTOR) and the Transport Accident Commission (TAC) claims data. VOTOR is a sentinel site registry which collects data about all orthopaedic trauma patients admitted for 24 h or more to four Victorian trauma‐receiving hospitals, including the two adult Level 1 trauma centres in the state. The TAC is a government owned no‐fault insurer that covers the costs of healthcare and ongoing costs of road transport‐related injuries in Victoria, Australia. The TAC covers total costs of injuries sustained in road transport‐related injuries including all medical, hospital, and rehabilitation costs in contrast to the Australian Medicare system and private healthcare system where gap payments are often required of the patient. Cost data was thus obtained from the TAC as it captures all costs claimed by the patient and healthcare providers for the management of patient injuries following a road traffic incident, with the data including itemised amounts for each billing encounter, in contrast to public healthcare data which includes only non‐itemised amounts and does not include any gap payments made by patients. This allows for detailed data to be obtained on initial admission, subsequent admission and rehabilitation costs, allowing for a more accurate representation of costs related to a particular injury. Ethics approval was obtained through the institutional Human Ethics Committee (Low Risk 73/19).

### 2.2 Procedures

Baseline demographic variables including patient age and gender were obtained from the medical record along with the management undertaken, whether operative or non‐operative. The medical record and initial admission discharge summary were screened for the presence of associated injuries and these were recorded along with ipsilateral upper limb injuries. VOTOR data was subsequently used to perform a data linkage with the TAC to obtain relevant cost data.

Cost data obtained from the TAC was matched with codes from the Medicare Benefit Schedule (MBS) pertaining to operative management of proximal humerus fractures (*codes presented in* Table S1). Cost data obtained from the TAC included the initial admission costs, subsequent acute hospital admission costs, and inpatient and outpatient rehabilitation costs. Acute admission costs included all costs of patient management post initial trauma until discharge. Subsequent admission costs were included from the TAC where a relevant MBS item number related to proximal humerus fracture management had been billed during an admission subsequent to the discharge date of the initial trauma admission. Rehabilitation costs were inclusive of both inpatient and outpatient rehabilitation and included costs for rehabilitation hospital stay, rehabilitation physicians and allied health including physiotherapy, occupational therapy, and social work. Cost data was inclusive of all care billed to the TAC by healthcare providers or claimed by the patient following their injury with a minimum follow up time of 2 years post initial injury. Cost data was adjusted for health inflation to the financial year 2021–2022 using data available from the Australian Institute of Health and Welfare [20].

### 2.3 Statistical Analysis

Statistical analysis was performed comparing two groups: patients who underwent initial operative management and patients managed non‐operatively. Comparison between groups using baseline patient and fracture characteristics was undertaken with student *t*‐test and Chi‐square test for normally distributed data and Mann–Whitney U test for non‐parametric data. Cost data was presented as both mean cost with standard deviation and median cost with interquartile range for each category. Cost data was a left‐skewed non‐parametric distribution and a comparison was undertaken using a robust estimation of Poisson distribution. Variables with a *p*‐value of 0.25 or less on the univariable analysis were included to account for potential confounders of the association between management group and cost. Statistical analysis was performed using STATA Version 17.0 (StataCorp LP, College Station, Texas, USA). Significance was set at *p* < 0.05.

## 3 Results

Of all proximal humerus fractures managed at the Level 1 trauma centre during the study period, a total of 112 were road transport‐related and had cost data available from the TAC. Of the 112 patients, 31 were managed non‐operatively and 81 were managed operatively. There were no differences in age or sex between the groups (Table 1).

**TABLE 1:** Patient demographics.

Factor	Non‐operative (*n* = 31)	Operative (*n* = 81)	*p*
Age (years), mean (SD)	50.1 (±20.3)	44.0 (±15.8)	0.10
Sex			0.70
Female	11 (35%)	32 (40%)	
Male	20 (65%)	49 (60%)	
Length of stay			
Mean (SD)	10.8 (11.9)	10.4 (11.5)	
Median (IQR)	4.7 (2.9–14.7)	6.8 (3.3–13.8)	0.69
Length of ICU stay			
Mean (SD)	4.5 (9.9)	0 (0–1)	
Median (IQR)	2.3 (5.6)	0 (0–0)	0.46
Number of other injuries			0.30
None	4 (13%)	19 (24%)	
1–2	13 (42%)	31 (38%)	
3 or more	14 (45%)	31 (38%)	
Ipsilateral upper limb injury			0.50
No	27 (87%)	66 (81%)	
Yes	4 (13%)	15 (19%)	
Discharge destination			0.07
Home	12 (39%)	34 (42%)	
Rehabilitation	17 (55%)	47 (58%)	
Death	2 (6%)	0 (0%)	

### 3.1 Total Treatment Cost

**FIGURE 1:**
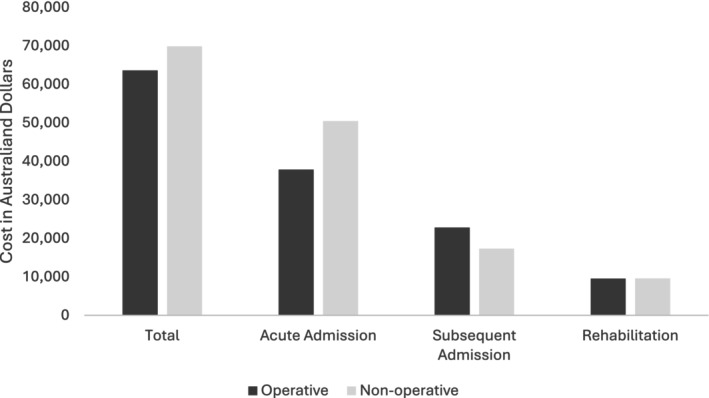
Comparison of mean treatment costs between groups.

**FIGURE 2:**
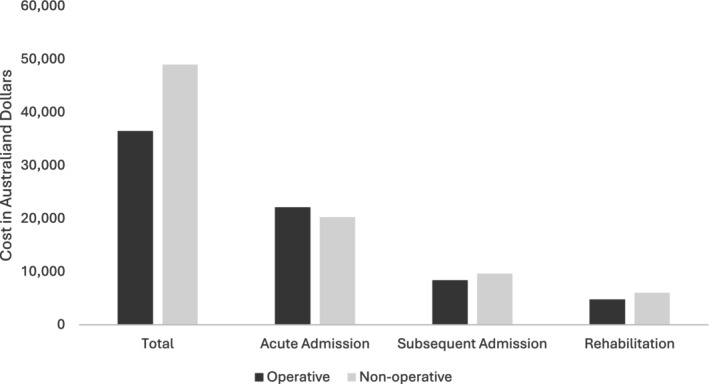
Comparison of median treatment costs between groups.

Mean total cost of management for the non‐operative group was A$69 883 (SD ±75 983) compared with a mean total management cost of A$63 615 (SD ±59 402) in the operative group (*p* = 0.65). The median total cost of management in the non‐operative group was A$48 982 (IQR 15 736–103 104) compared with a median total management cost in the operative group of A$36 457 (IQR 19 893–93 659) (*p* = 0.99). Mean treatment costs and median treatment costs are represented in Figures 1 and 2 respectively.

### 3.2 Acute Admission Cost

When including only costs incurred during the initial admission episode, the mean costs of non‐operative management were A$50 461 (SD ±67 950) compared with A$37 850 (SD ±38 805) for patients that underwent operative management (*p* = 0.22). The median costs of the two respective groups for the acute admission were A$20 261 (IQR 9655–49 640) in the non‐operative group and A$22 111 (IQR 15 532–45 946) in the operative group (*p* = 0.65).

### 3.3 Subsequent Admission Cost

The mean subsequent admission cost was A$17 320 (SD ±20 512) in the non‐operative group and A$22 803 (SD ±33 126) in the operative group (*p* = 0.47). The median subsequent admission cost was A$9 643 (IQR 3641–24 392) in patients that underwent non‐operative management and A$8378 (IQR 1320–28 702) in patients that underwent operative treatment (*p* = 0.77).

### 3.4 Rehabilitation Cost

Total rehabilitation costs for inpatient and outpatient rehabilitation were a mean of A$9609 (SD ±12 723) in the non‐operatively managed cohort compared to A$9549 (SD ±13 082) in the operatively managed cohort (*p* = 0.99). The median rehabilitation costs were A$6016 (IQR 463–13 318) in the non‐operative cohort and A$4754 (IQR 715–12 227) in the non‐operative cohort (*p* = 0.95).

## 4 Discussion

This study reports on healthcare costs of managing proximal humerus fractures in an Australian population. The findings of this study suggest that there is no difference in healthcare costs regardless of the pathway undertaken in managing proximal road transport‐related humerus fractures. Notably, there was no difference in total costs between operative and non‐operative management, and this was also the case when costs were subdivided into acute admission costs, subsequent admission costs, and rehabilitation costs.

The findings of this study are in contrast to previous studies performed in Europe and the United States of America where patients undergoing operative management had significantly higher costs than patients managed non‐operatively [11, 13, 14, 15, 16, 18]. London et al. demonstrated in their study of an American cohort of patients a substantially higher cost associated with operative intervention in the form of internal fixation (approximately US$10 000 more expensive than non‐operative management) and shoulder arthroplasty (approximately US$20 000 more expensive than non‐operative management) [18]. Cheng et al. found in a cohort of American geriatric proximal humerus fractures a median increase in hospital costs with surgical treatment of US$10 684 compared with non‐surgical treatment; however, those that underwent surgical fixation were more likely to be discharged home [16]. Notably, in their study, both surgical and non‐surgical cohorts demonstrated similar complication rates which is in contrast to the majority of literature demonstrating high complication rates with surgical management of proximal humerus fractures [7, 8, 9, 21, 22, 23, 24, 25].

This study demonstrated high subsequent admission costs for the management of proximal humerus fractures in both management groups. Prior studies have focused on the high complication rates following surgical management of these injuries, with failure rates of locking plate fixation consistently demonstrated to be greater than 20%–30% [8, 9, 23, 24, 26]. There is less evidence available in non‐operative management, however recent studies have demonstrated complication rates as high as 75% following non‐operative intervention [22, 23]. The high complication rates demonstrated in these injuries in prior studies likely contribute to the high subsequent admission costs found in this study, however our study was not designed to determine the causation of these costs. There is limited other evidence available on the in‐hospital costs secondary to readmission following these injuries. In the United States of America, Thorsness et al. demonstrated a large increase of mean in‐hospital costs of US$54 345 when readmission occurred for the management of a proximal humerus fracture, adding a significant burden to healthcare systems [17]. The increased cost found in that study is substantially higher than that in our cohort, however the studies have been conducted in different healthcare systems and patient populations and are thus not directly comparable. Notably in our cohort there was no difference in cost between intervention groups where subsequent admissions occurred, suggesting that cost should not influence the initial management decision, rather optimising the care provided should be the focus to minimise the rate of subsequent admissions and associated healthcare costs.

Prior literature has predominantly focused on acute admission costs and the costs of initial care with no inclusion or differentiation of ongoing rehabilitation costs. This study has demonstrated no difference between rehabilitation costs in operatively and non‐operatively managed patients. Mean rehabilitation costs were high in both groups (A$9609 in the non‐operatively managed cohort compared to A$9549 in the operative cohort), which incorporated both inpatient and outpatient costs. The high rehabilitation costs are partly attributable to the multiple injuries that the patients in this study sustained, and thus the inclusion of additional rehabilitation required for those injuries, as opposed to solely being related to the proximal humerus fracture management. Despite this limitation, the finding of no difference in costs between the groups does not support selecting a particular management option to expedite the rehabilitation process or to minimise the associated costs. No prior studies identified have included isolated rehabilitation costs incurred post proximal humerus fracture. Previous evidence has focused solely on discharge destination and length of stay, with improved rates of discharge home and shorter lengths of stay with operative management; however, these findings did not correlate with reduced cost of management in those studies [13, 16]. In contrast, our cohort demonstrated no difference in length of stay or discharge destination following either management pathway.

Whilst the strengths of this study include the comprehensive nature of the cost data for the management of proximal humerus fractures due to the funding model of the TAC, there are several limitations. Firstly, the overall numbers in this study are relatively small in comparison to prior published literature despite the study time period. This is in part due to the use of a single site along with the use of the TAC scheme being limited to patients injured in road traffic incidents. Furthermore, the use of an administrative database, which is dependent on the quality and accuracy of the codes entered, is subject to errors in coding. In the case of those undergoing operative management, the risk of coding errors was minimised in this study by the pairing of cost data with the associated MBS surgical codes, ensuring costs were associated with accurate coding that had been performed by the treating surgeon. The use of the TAC scheme, whilst a comprehensive database of costs, is limited in that it only captures costs billed to the TAC by healthcare providers or claimed by patients, and thus there is a potential for some costs to not have been captured if patients self‐funded costs and did not claim from the TAC. This is likely to be a negligible proportion of the costs involved in care. Another limitation of this study is the high proportion of patients who had sustained multiple injuries, increasing the acute hospital costs secondary to including costs incurred in managing all injuries in addition to the management of the proximal humerus fracture. This was not the case for subsequent admissions as these were only included if associated with the relevant MBS code for the management of a proximal humerus fracture. Although the cost data in the acute admission was unable to be isolated to only the cost of managing the proximal humerus fracture, there was no difference between groups in number of injuries sustained. Thus, whilst total costs were higher than expected due to the multiple trauma sustained, the groups were comparable and the findings of no difference in costs between groups remain representative of the costs incurred with either operative or non‐operative management. Lastly, the use of TAC data in a road‐traffic incident population limits the generalisability of the findings due to the difference in funding models between the Medicare public healthcare system, private healthcare system, and the TAC.

## 5 Conclusion

This study found no difference in acute in‐hospital costs, subsequent admission costs, or rehabilitation costs in Australian patients injured in road‐traffic incidents with traumatic proximal humerus fractures managed either operatively or non‐operatively, in a Level 1 trauma hospital setting. These findings are in contrast to studies performed in Europe and North America. Notably, regardless of management undertaken, there were high costs associated with subsequent admissions. The findings of this study suggest that cost should not be a factor in the decision‐making process when considering operative or non‐operative management of proximal humerus fractures in patients injured in road‐traffic incidents compensated by the TAC. The high subsequent admission costs suggest a need to optimise the management of proximal humerus fractures to reduce the complication rate and requirement for further surgery.

## Funding

VOTOR is funded by the Transport Accident Commission.

## Conflicts of Interest

The authors declare no conflicts of interest.

## Supporting information


**Table S1:** Matched MBS codes with item descriptors.

## Data Availability

The data that support the findings of this study are available on request from the corresponding author. The data are not publicly available due to privacy or ethical restrictions.
