# Supplementary material for: A Comparison of the Costs of Managing Proximal Humerus Fractures in a Cohort of Patients Injured in Road Traffic Incidents Under the Transport Accident Commission Scheme in the State of Victoria
Source: ANZ J Surg. 2026 Apr 9;96(6):1594–9. doi: 10.1111/ans.70669 (PMC13327594; doi:10.1111/ans.70669)
Supplement: Supplementary file 1 — Table S1: Matched MBS codes with item descriptors. [file ANS-96-1594-s001.docx]

Supplementary Table 1: Matched MBS codes with item descriptors

| **MBS Code** | **Descriptor** |
| --- | --- |
| 43530 | Operation on scapula, ulna, radius, tibia, fibula, humerus or femur, by open or arthroscopic means, for septic arthritis or osteomyelitis |
| 47009 | Treatment of dislocation of shoulder, requiring general anaesthesia, other than a service to which item 47012 applies |
| 47012 | Treatment of dislocation of shoulder, requiring general anaesthesia, by open reduction |
| 47411 | Treatment of fracture of tuberosity of humerus |
| 47414 | Treatment of fracture of tuberosity of humerus, by open reduction |
| 47417 | Treatment of fracture of tuberosity of humerus and associated dislocation of shoulder, by closed reduction |
| 47420 | Treatment of fracture of tuberosity of humerus and associated dislocation of shoulder, by open reduction |
| 47423 | Humerus, proximal, treatment of fracture of, other than a service to which item 47426, 47429 or 47432 applies |
| 47426 | Humerus, proximal, treatment of fracture of, by closed reduction |
| 47429 | Humerus, proximal, treatment of fracture of, by open reduction |
| 47432 | Humerus, proximal, treatment of intra‑articular fracture of, by open reduction |
| 47435 | Humerus, proximal, treatment of fracture of, and associated dislocation of shoulder, by closed reduction |
| 47438 | Humerus, proximal, treatment of fracture of, and associated dislocation of shoulder, by open reduction |
| 47441 | Humerus, proximal, treatment of intra-articular fracture of, and associated dislocation of shoulder, by open reduction |
| 47924 | Removal of one or more buried wires, pins or screws (inserted for internal fixation purposes), with incision, other than a service associated with a service to which item 47927 or 47929 applies—one bone |
| 47927 | Removal of one or more buried wires, pins or screws (inserted for internal fixation purposes)—one bone |
| 47929 | Removal of fixation elements (including plate, rod or nail and associated wires, pins, screws or external fixation), other than a service associated with a service to which item 47924 or 47927 applies—one bone |
| 48412 | Osteotomy of humerus, without internal fixation |
| 48415 | Osteotomy of humerus, with internal fixation |
| 48900 | Shoulder, excision of coraco‑acromial ligament or removal of calcium deposit from cuff or both |
| 48903 | Shoulder, decompression of subacromial space by acromioplasty, excision of coraco‑acromial ligament and distal clavicle, or any combination |
| 48906 | Shoulder, repair of rotator cuff, including excision of coraco‑acromial ligament or removal of calcium deposit from cuff, or both—other than a service associated with a service to which item 48900 applies |
| 48909 | Shoulder, repair of rotator cuff, including decompression of subacromial space by acromioplasty, excision of coraco‑acromial ligament and distal clavicle, or any combination, other than a service associated with a service to which item 48903 applies |
| 48915 | Shoulder, hemi‑arthroplasty of |
| 48918 | Anatomic or reverse total shoulder replacement, including any of the following (if performed): (a) associated rotator cuff repair; (b) biceps tenodesis; (c) tuberosity osteotomy; |
| 48921 | Shoulder, total replacement arthroplasty, revision of |
| 48924 | Revision of total shoulder replacement, including either or both of the following (if performed): (a) bone graft to humerus; (b) bone graft to scapula |
| 48927 | Shoulder prosthesis, removal of |
| 48939 | Shoulder, arthrodesis of, with synovectomy if performed |
| 48942 | Arthrodesis of shoulder, with bone grafting or internal fixation, including either or both of the following (if performed): (a) removal of prosthesis; (b) synovectomy; other than a service associated with a service to which item 48245, 48248, 48251, 48254 or 48257 applies |
| 48945 | SHOULDER, diagnostic arthroscopy of (including biopsy) - not being a service associated with any other arthroscopic procedure of the shoulder region |
| 48948 | SHOULDER, arthroscopic surgery of, involving any 1 or more of: removal of loose bodies; decompression of calcium deposit; debridement of labrum, synovium or rotator cuff; or chondroplasty - not being a service associated with any other arthroscopic procedure of the shoulder region |
| 48951 | SHOULDER, arthroscopic division of coraco-acromial ligament including acromioplasty - not being a service associated with any other arthroscopic procedure of the shoulder region |
| 48954 | Synovectomy of shoulder, performed as an independent procedure, including release of contracture (if performed), other than a service associated with a service to which another item in this Schedule applies if the service described in the other item is for the purpose of  performing a procedure on the shoulder region by arthroscopic means |
| 48958 | Joint stabilisation procedure for multi-directional instability of shoulder, anterior or posterior repair, by open or arthroscopic means,  including labral repair or reattachment (if performed), excluding bone grafting and removal of hardware, other than a service associated with a service to which another item in this Schedule applies if the service described in the other item is for the purpose of  performing a procedure on the shoulder region by arthroscopic means |
| 48960 | SHOULDER, reconstruction or repair of, including repair of rotator cuff by arthroscopic, arthroscopic assisted or mini open means; arthroscopic acromioplasty; or resection of acromioclavicular joint by separate approach when performed - not being a service associated with any other procedure of the shoulder region |
| 48980 | Excision of heterotopic ossification, myositis ossificans or post-traumatic ossification in the shoulder girdle |
